# i-rDNA: alignment-free algorithm for rapid *in silico* detection of ribosomal gene fragments from metagenomic sequence data sets

**DOI:** 10.1186/1471-2164-12-S3-S12

**Published:** 2011-11-30

**Authors:** Monzoorul Haque Mohammed, Tarini Shankar Ghosh, Sudha Chadaram, Sharmila S Mande

**Affiliations:** 1 Bio-Sciences R&D Division, TCS Innovation Labs, Tata Consultancy Services Limited, 1 Software Units Layout, Hyderabad 500 081, Andhra Pradesh, India

## Abstract

**Background:**

Obtaining accurate estimates of microbial diversity using rDNA profiling is the first step in most metagenomics projects. Consequently, most metagenomic projects spend considerable amounts of time, money and manpower for experimentally cloning, amplifying and sequencing the rDNA content in a metagenomic sample. In the second step, the entire genomic content of the metagenome is extracted, sequenced and analyzed. Since DNA sequences obtained in this second step also contain rDNA fragments, rapid *in silico* identification of these rDNA fragments would drastically reduce the cost, time and effort of current metagenomic projects by entirely bypassing the experimental steps of primer based rDNA amplification, cloning and sequencing. In this study, we present an algorithm called i-rDNA that can facilitate the rapid detection of 16S rDNA fragments from amongst millions of sequences in metagenomic data sets with high detection sensitivity.

**Results:**

Performance evaluation with data sets/database variants simulating typical metagenomic scenarios indicates the significantly high detection sensitivity of i-rDNA. Moreover, i-rDNA can process a million sequences in less than an hour on a simple desktop with modest hardware specifications.

**Conclusions:**

In addition to the speed of execution, high sensitivity and low false positive rate, the utility of the algorithmic approach discussed in this paper is immense given that it would help in bypassing the entire experimental step of primer-based rDNA amplification, cloning and sequencing. Application of this algorithmic approach would thus drastically reduce the cost, time and human efforts invested in all metagenomic projects.

**Availability:**

A web-server for the i-rDNA algorithm is available at http://metagenomics.atc.tcs.com/i-rDNA/

## Background

The majority of microorganisms present in natural ecosystems cannot be cultured in the laboratory and hence remain unexplored. To exploit the potential of this unexplored diversity, the new field, called metagenomics, has been initiated in the recent years.

The first step in a typical metagenomics project involves estimating the microbial diversity present in the environmental sample under study. Obtaining accurate estimates of this diversity is an important step and forms the first objective of any metagenomics project. Analyzing 16S ribosomal RNA (16S rRNA) gene (commonly referred to as 16S rDNA) sequences is the most commonly used method for rapidly estimating microbial diversity [1,2]. This method is based on the following premise. Major portions of the 16S rDNA sequence are highly conserved across all bacterial and archaeal species [3]. Using primers that can hybridize with these conserved portions, 16S rDNA sequences of most of the microbes (both culturable and un-culturable) in a given environmental sample are directly amplified, cloned and sequenced. Subsequently, the taxonomic affiliation of the obtained 16S rDNA sequences is identified using 16S rDNA sequence analysis platforms such as Greengenes [4], RDP classifier [5], etc. Enumerating the number of 16S rDNA sequences assigned to various taxonomic clades helps in quantifying the relative abundance of various organisms/taxa present in the given environmental sample.

In the second step of any metagenomics project, the entire genomic content of the environmental sample under study is extracted and sequenced. Millions of DNA sequences originating from the genomes of various microbes in the sample are thus obtained. Using computational techniques, the genes harbored in these DNA sequences are identified and functionally characterized.

Since the first step, i.e the 16S rDNA profiling, is expected to provide a near comprehensive snapshot of microbial diversity, almost all metagenomic projects spend considerable amount of time, money and manpower for completing the various experimental procedures involved in this step. For instance, in a metagenomics study performed by Manichanh *et al.* (2008), the process of 16S rDNA extraction and amplification (for a relatively small sample of just 50,000 sequence clones) took approximately 6 months and required three persons and the cost incurred was more than $70,000 of materials and equipments [6].

Since the sequences obtained from the complete DNA content of an environmental sample (in the second step mentioned above) contains rDNA fragments, it is possible (in theory) to computationally identify these rDNA fragments directly from the sequenced environmental DNA. Considering the availability of faster and cheaper sequencing technologies, such as 454 [7], the applicability of an alternative *in silico* approach to identify 16S rDNA fragments directly from the completely sequenced metagenomes will be immense, since it would not only save huge amount of time, efforts and money (by bypassing the entire experimental step of primer based 16S rDNA amplification, cloning and sequencing), but would also circumvent various experimental limitations associated with 16S rDNA profiling [8-10]. The recently published 'meta-rna' program [11] represents a fairly successful attempt in developing such an alternative *in silico* approach. As a pre-processing step, this program uses the HMMER program [12] for first building a set of HMMs (Hidden Markov Models) that reflect/represent the sequence conservation found within rDNA sequences in archaeal and bacterial clades. During run time, the program aligns sequences in a given metagenomic data set against these precomputed HMMs, and sequences showing significant alignment scores (in terms of e-value) to these models are identified by the meta-rna program as 16S rDNA fragments.

In spite of having significantly high detection sensitivity (even with metagenomic sequences as short as 100 bp), enormous compute time is needed by the meta-rna program for analyzing huge metagenomic data sets. Our experiments (on a desktop having 2 GB RAM and 2.33 GHz dual-core processor) with simulated data sets indicated that the meta-rna program takes approximately 19, 49, 84 and 156 milliseconds for analyzing a query sequence of length 100, 250, 400, and 800 base pairs (bp), respectively. At this rate, approximately 325 hours (i.e. greater than 13 days) would be needed for analyzing the 7,521,215 sequences (average length around 800 bp) constituting the Global Ocean Sampling Expedition Microbial Metagenomic data sets [13-15]. Even for a relatively smaller data set, such as the mouse gut metagenome, consisting of 1,744,283 sequences with average length around 100 bp [16], the total analysis time needed by the meta-rna program would exceed 9 hours.

A careful examination of the analysis procedure reveals that the meta-rna program needs to analyze every individual sequence in a given metagenomic data set to identify rDNA fragments. Besides being time consuming, analyzing every sequence is also not an ideal procedure (especially in this case) given the following observation. Several recent studies on metagenomic data sets obtained from diverse environments such as Sargasso sea [13], soil [17] and sludge [18] have indicated that the percentage of metagenomic sequences that harbor portions of the rDNA gene is generally less than 0.2%. In this paper, we propose an approach termed as i-rDNA (identification of ribosomal DNA) that can rapidly identify a small subset of metagenomic sequences (from amongst all sequences constituting the complete data set) that, in high probability, harbor portions of the rDNA gene. Sequences belonging to only this (small) subset can further be analyzed using the meta-rna program. We demonstrate that adopting the i-rDNA program as a precursor step to the meta-rna program reduces the overall detection time by several fold. Importantly, this significant reduction in analysis time does not affect the overall detection sensitivity.

## Results

### i-rDNA algorithm

The i-rDNA algorithm is based on the following premise. Major portions of the 16S rDNA gene sequence are highly conserved across all bacterial and archaeal species. Consequently, 16S rDNA sequences are expected to have an oligonucleotide composition distinct as compared to the oligonucleotide composition of DNA sequences originating from other portions of the genome. Therefore if genome fragments obtained from all known bacterial and archaeal genomes are clustered based on oligonucleotide usage patterns, fragments harboring portions of the 16S rDNA gene sequence (irrespective of their taxonomic origin) will get spatially localized to a 'few' clusters in feature vector space with high frequency. In the pre-processing step, the method presented in this paper identifies these 'few' clusters (amongst all clusters in feature vector space) and tags them as 'probable' rDNA clusters. While performing the actual analysis (i.e during actual run time), the i-rDNA algorithm first identifies a set of clusters (amongst all clusters) whose sequences have an oligonucleotide composition similar to that of the query sequence being analyzed. Subsequently, i-rDNA algorithm classifies a given query sequence as a 'probable 16S rDNA fragment' if the overlap between the set of clusters (identified as compositionally similar to that of the query sequence) and the set of clusters pre-tagged as 'probable 16S rDNA clusters' exceeds a pre-determined threshold percentage. Given that the run time steps of the i-rDNA algorithm only involves finding the closest clusters (in terms of oligonucleotide composition) for a given query sequence, and subsequently checking if the overlap percentage (between the closest clusters and the pre-tagged 'probable 16S rDNA clusters') exceeds a pre-determined threshold, the i-rDNA algorithm is able to rapidly analyze huge metagenomic data sets within a short span of time and identify (a small set of) probable rDNA fragments from amongst millions of sequences constituting the complete metagenomic data set. The meta-rna algorithm can then be employed for analyzing this small set of candidate 16S rDNA query fragments.

**Figure 1:**
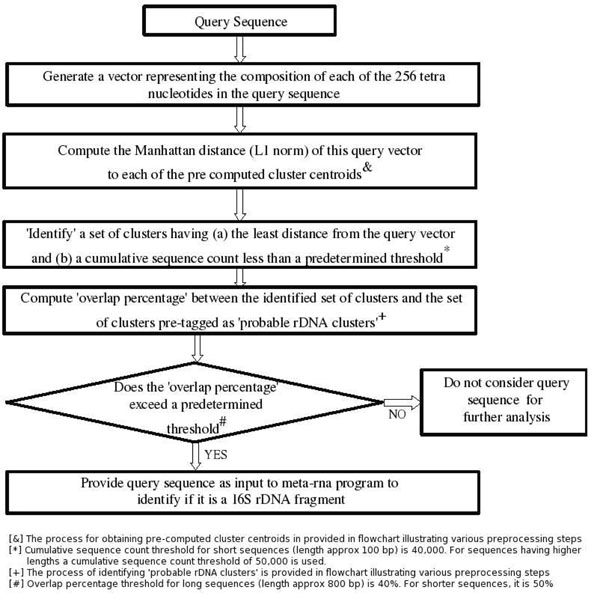
**Steps of the i-rDNA approach** Flowchart illustrating the various steps followed by the i-rDNA approach.

Figure 1 illustrates the various steps of the i-rDNA approach (described above). Details of the procedure followed for (a) pre-clustering bacterial and archaeal genome fragments, (b) identifying and pre-tagging a subset of clusters as 'probable 16S rDNA clusters' and (c) determining the value of the 'overlap percentage' threshold are given in methods section.

### Validation

Twelve simulated metagenomic data sets were used for evaluating the performance of the i-rDNA algorithm. Three of these data sets (referred to as simLC-Sanger, simMC-Sanger and simHC-Sanger) were downloaded from http://fames.jgi-psf.org/. These simulated data sets of varying taxonomic complexity (Low, Medium and High) were constructed by combining real time sequencing reads from 113 organisms listed in Additional File 1. These simulated 'gold-standard' data sets [19] are generally used for evaluating the performance of algorithms used in metagenomic analysis. Using the same coverage values used while creating these 3 simulated data sets (having real time reads from Sanger sequencing technology), we applied the program MetaSim [20] on the same 113 organisms for generating 9 more data sets. These additional data sets can be divided into three groups having sequences of average lengths 400, 250 and 100 base pairs respectively. Sequences in these data sets thus simulated the typical sequence lengths obtained from existing sequencing technologies, namely 454-Titanium (sequence length centered around 400 bp), 454-Standard (250 bp), and Roche-GS20 (100 bp).

These 12 simulated data sets were first given as input to the meta-rna algorithm. The number of rDNA fragments identified in each data set by the meta-rna algorithm was noted down. The same data sets were then given as input to the i-rDNA algorithm. Sequences identified by the i-rDNA algorithm as 'probable' 16S rDNA fragments (in each data set) were consequently given as input to the meta-rna algorithm. The number of rDNA fragments identified in this two step procedure (involving both i-rDNA and meta-rna) and the overall analysis time taken for this alternative approach was noted down. Results of these experiments are given in Table 1.

**Table 1:** Results indicating detection sensitivity and fold-reduction in search-space achieved using the i-rDNA algorithm.

Validation data set	Total number of sequences (X)	i-rDNA predicted 'probable 16S rDNA' sequences (Y)	Fold reduction in search space (X/Y)	meta-RNA predicted 16S rDNA sequences (A)	i-rDNA predicted 16S rDNA sequences within 'A' (B)	Detection sensitivity (B*100/A)
SimLC -Sanger	97493	9262	10.5	183	156	85.2
SimMC -Sanger	114456	10873	10.5	268	236	88.1
SimHC -Sanger	116770	10505	11.1	392	341	87.0



SimLC-454-400	224422	30663	7.3	268	241	89.9
SimMC-454-400	268350	36145	7.4	337	312	92.6
SimHC-454-400	267076	37492	7.1	452	407	90.0



SimLC-454-250	359076	53795	6.7	404	374	92.6
SimMC-454-250	429360	65498	6.6	506	476	94.1
SimHC-454-250	427321	64922	6.6	679	637	93.8



SimLC-454-100	897689	153505	5.8	845	776	91.8
SimMC-454-100	1073401	174535	6.2	1035	971	93.8
SimHC-454-100	1068303	130974	8.2	1514	1371	90.6

* X = The total number of sequences in a given data set; Y = Total number of sequences predicted by the i-rDNA program as 'probable rDNA sequences' in that data set

** A = The total number of 16S rDNA sequences predicted by meta-rna program in the entire data set;

B = Number of 16S rDNA sequences within the subset of 'probable r-DNA sequences' predicted by i-rDNA

The overall pattern of results indicates that the two-step procedure (i-rDNA followed by the meta-rna) is able to detect around 85-94% of 16S rDNA fragments in a given data set. Around 9-17% of sequences in the data sets are observed to be predicted by the i-rDNA algorithm as 'probable 16S rDNA sequences'. As expected, providing this relatively small subset of 'probable' sequences as input to the meta-rna algorithm (instead of the whole input data set) results in 6-11 fold reduction in the overall processing time. It is significant to note that negligible time is required by the i-rDNA algorithm for identifying the initial 16S rDNA candidate set. The i-rDNA algorithm is able to process approximately 35,000 sequences/minute. For example, i-rDNA algorithm processed 1,744,283 mouse gut metagenome sequences [16] in 50 minutes, and in the process it identified 180,795 'probable' 16S rDNA sequences. Another 56 minutes were needed by the meta-rna algorithm for analyzing this subset of probable candidates identified by i-rDNA. The overall processing time for this data set was thus reduced from 552 minutes (~9 hours) taken by meta-rna to 106 minutes (less than 2 hours) by i-rDNA followed by meta-rna. A similar estimate on the 7,521,215 sequences in the Global Ocean Sampling Expedition Microbial Metagenomic data sets [13-15] indicates a significant reduction in total analysis time from approximately 325 hours (>13 days) to 34 hours (< 2 days).

Given that the final objective of 'computationally' identifying 16S rDNA fragments is to obtain a taxonomic profile of any given metagenomic data set, any loss in detection sensitivity by the i-rDNA approach will be 'acceptable', only if the 'reduced detection sensitivity' does not significantly alter the taxonomic profile of a given metagenomic data set. Using a recently published metagenomic data set, the following experiment was performed to verify if 'similar' taxonomic profiles are obtained by providing input sequences to (1) i-rDNA or (2) directly to the meta-RNA program. Approximately 1.3 million sequences constituting the metagenomic data set sampled from a malnourished child [21] were provided as input to (1) i-rDNA and (2) directly to the meta-RNA program. Providing the entire data set directly to the meta-RNA program identified 3276 16S rDNA sequences. On the other hand, the i-rDNA program (in conjunction with the meta-RNA program) identified 2678 16S rDNA sequences (approximately 82% of 3276 sequences). The RDP classifier [5] was subsequently used for obtaining the taxonomic profile of sequences identified as 16S rDNA fragments by either methods. The taxonomic profiles obtained were then compared at various taxonomic levels.

Results of the above experiments (summarized in Table 2) indicate that the taxonomic profiles (in terms of groups identified along with their relative percentages) obtained using 16S sequences predicted by either methods are very similar. Consequently, the marginal loss in detection sensitivity of the i-rDNA program does not seem to impact the obtained taxonomic profile. Moreover, the time taken by the i-rDNA approach for analysing this metagenome was observed to be approximately 6 times lesser (4.3 hours) as compared to that of the meta-RNA program (25.3 hours). The above results reaffirm the suitability/applicability of the i-rDNA program for analysing real metagenomic data sets.

**Table 2:** Comparison of the taxonomic assignments^*^ of 16S sequences (identified by i-rDNA and the meta-RNA program) in the malnourished child gut metagenome^#^ at the taxonomic levels of (A) Order (B) Class and (C) Phylum.

(A) Order	16S sequences identified by		Percentage of 16S sequences identified by	
	
	i-rDNA	meta-RNA	i-rDNA	meta-RNA
Bacteroidales	1456	1469	60.9	59.5
Campylobacterales	247	286	10.3	11.6
Clostridiales	208	208	8.7	8.4
Fusobacteriales	143	182	6.0	7.4
Aeromonadales	143	130	6.0	5.3
Enterobacteriales	78	78	3.3	3.2
Burkholderiales	78	78	3.3	3.2
Mycoplasmatales	13	13	0.5	0.5
Erysipelotrichales	13	13	0.5	0.5
Bifidobacteriales	13	13	0.5	0.5

**TOTAL**	**2392**	**2470**		

**(B) Class**	**16S sequences identified by**		**Percentage of 16S sequences identified by**	
	
	**i-rDNA**	**meta-RNA**	**i-rDNA**	**meta-RNA**

Bacteroidia	1456	1469	60.5	59.2
Epsilonproteobacteria	260	286	10.8	11.5
Gammaproteobacteria	221	221	9.2	8.9
Clostridia	208	208	8.6	8.4
Fusobacteria	143	182	5.9	7.3
Betaproteobacteria	78	78	3.2	3.1
Mollicutes	13	13	0.5	0.5
Erysipelotrichi	13	13	0.5	0.5
Actinobacteria	13	13	0.5	0.5

**TOTAL**	**2405**	**2483**		

**(C) Phylum**	**16S sequences identified by**		**Percentage of 16S sequences identified by**	
	
	**i-rDNA**	**meta-RNA**	**i-rDNA**	**meta-RNA**

Bacteroidetes	1508	1599	61.1	60.3
Proteobacteria	572	624	23.2	23.5
Firmicutes	221	221	8.9	8.3
Fusobacteria	143	182	5.8	6.9
Tenericutes	13	13	0.5	0.5
Actinobacteria	13	13	0.5	0.5

**TOTAL**	**2470**	**2652**		

* The taxonomic affiliations were obtained by providing the sequences as input to the RDP classifier (Wang Q, Garrity GM, Tiedje JM, Cole JR. Naive Bayesian classifier for rapid assignment of rRNA sequences into the new bacterial taxonomy. *Appl Environ Microbiol*. 2007 73(16):5261-7.

# Gupta SS, Mohammed MH, Ghosh TS, Kanungo S, Nair GB, Mande SS. Metagenome of the gut of a malnourished child. *Gut Pathog.* 2011 May 20;3:7.

To further demonstrate the applicability of the i-rDNA algorithm for typical metagenomic data sets (wherein a majority of sequences originate from new or hitherto unknown taxonomic clades), it is important to test the present algorithm's ability in detecting the rDNA fragments originating from organisms belonging to new (or hitherto unknown) taxonomic clades. For this purpose, the following 'leave one clade out' testing strategy was adopted. In an iterative manner, 16S rDNA sequences belonging to a species or genus or family or order or class or phylum were not considered while pre- computing the 'probable 16S rDNA clusters' (section B in methods). These six different simulated scenarios were referred to as 'NEW SPECIES', 'NEW GENUS', 'NEW FAMILY', 'NEW ORDER', 'NEW CLASS' and 'NEW PHYLUM', respectively. Subsequently, these left out sequences (which now simulate sequences from an unknown species or genus or family or order or class or phylum respectively) were tested against the respective modified set of 'probable 16S rDNA clusters'. These tests were also carried out using four sequence lengths (100, 250, 400 and 800 bp) that mimicked sequences obtained using the four commonly used sequencing technologies mentioned above.

Table 3 shows the detection sensitivity of the i-rDNA algorithm with 16S rDNA fragments originating from new species, genus, family, order, class and phylum. Results indicate that, for query sequences having lengths greater than 250 bp, the i-rDNA algorithm is able to detect fragments of 16S rDNA originating from new organisms (belonging to even an entirely new phylum) with greater than 75% sensitivity. Even with the weak composition signal obtained from sequences with length as low as 100 bp, the detection sensitivity of the i-rDNA algorithm is observed to be greater than 70%. This indicates that the i-rDNA algorithm can be used for detecting 16S rDNA fragments from typical metagenomic data sets, wherein majority of organisms belong to hitherto unknown species, genus, family, order, class and phyla.

**Table 3:** The performance of i-rDNA algorithm in six different simulated metagenomic scenarios.

Source of the query sequence	Percentage of 16S rDNA gene fragments correctly identified by i-rDNA algorithm			
	
	Length of the query sequences			
	
	800 bp	400 bp	250 bp	100 bp
**New species**	96.4	94.1	91.3	85.3
**New genus**	94.5	91.3	85.3	71.2
**New family**	93.5	90.2	84.1	70.6
**New order**	92.3	90.1	84.8	70.6
**New class**	91.0	90.0	83.8	70.0
**New phylum**	89.2	88.4	82.4	69.5

## Discussion

A typical metagenomic analysis comprises of two major phases of experimentation. The first phase involves isolation, amplification and sequencing of the rDNA content of the environmental sample to obtain estimates of taxonomic diversity. In the second phase, the entire genomic content of the environmental sample is sequenced and analyzed. Both phases therefore involve costs with respect to time, money and manpower for carrying out experimentation and analysis. Given the ability of the approach (i.e using i-rDNA algorithm in conjunction with the meta-rna program) to accurately and rapidly identify rDNA fragments directly from the sequenced genomic content (obtained in the second phase), the *in silico* approach suggested in this paper has the potential to completely bypass the first phase of experimentation, thereby eliminating the costs associated with this phase.

It should be noted that conservation of oligonucleotide usage patterns within rDNA genes forms the premise of the i-rDNA algorithm. Consequently, the subset of sequences predicted by the i-rDNA algorithm as probable rDNA fragments is a mix of true 16S rDNA fragments and other genomic fragments (from non-rDNA gene regions) sharing an oligonucleotide composition similar to the rDNA genes. However, the size of the predicted subset (by i-rDNA) as a proportion of the total data set is observed to be around 10-15%. This indicates that the oligonucleotide composition of approximately 10-15% of genomic regions in prokaryotic organisms resembles that of the 16S rDNA gene. This region is probably well conserved and would be interesting for further analysis. However it should be noted that the subsequent use of the meta-rna program of this predicted subset helps in identifying the true set of rDNA fragments in the given data set. As demonstrated in this paper, using the meta-rna program on the subset of the sequences (predicted by i-rDNA), rather than the whole data set results in significant saving of time with minimal loss in detection sensitivity.

Furthermore, given the premise of the i-rDNA algorithm is based on conservation of oligonucleotide usage patterns, the i-rDNA approach can, in principle, be applied for the identification of any phylogenetic marker gene exhibiting universal sequence conservation at nucleotide level. The current study involving 16S rDNA sequences (as marker genes) can be taken as a proof of concept for the above assertion. However, the following aspects need to be considered before extending the i-rDNA approach to other phylogenetic marker genes. First, the marker gene should be conserved across all phylogenetic clades. Second, sequences belonging to the marker gene should also be available in sufficient numbers. This is important given that the training process (in the i-rDNA approach) involves identification of a set of ‘probable marker gene clusters’. The higher the number of marker gene sequences available, the more robust is the training process.

The initial step in the i-rDNA approach involves (a one-time) pre-clustering of genomics fragments generated from completely sequenced microbial genomes. In the present study, the time taken for performing this initial step was only about 3 hours on a simple desktop (2.33 GHz Intel dual core processor, 2GB RAM). This includes the time taken for (1) generating all the vectors (2) the actual clustering step, as well as, for (3) obtaining the vectors corresponding to the final cluster centroids. However, it should be noted that the initial clustering step (as well as the steps followed by i-rDNA for identifying a set of ‘probable rDNA clusters’) is a 'one-time' process. The actual run-time steps of the i-rDNA approach do not involve a repetition of the initial clustering steps.

**Figure 2:**
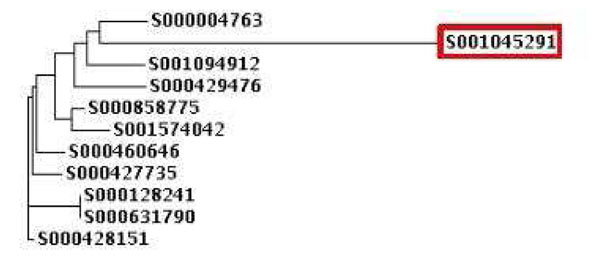
**Analysis of a 16S rDNA sequence missed by meta-RNA program.** A ClustalW tree depicting a 16S rDNA sequence (Sequence Id: S001045291) from the species *Mycobacterium sp.* 1764 as outlier compared to other sequences from the genus Mycobacterium. The meta-RNA program misses this sequence because of its’ distinct sequence architecture.

The final detection of 16S rDNA fragments within the i-rDNA approach is dependent on the detection sensitivity of the downstream meta-RNA program. Results in the original published paper [11] of the meta-RNA program indicate high detection sensitivity. Even in our experiments, the percentage of 16S sequences missed by the meta-RNA program was observed to be negligible. For instance, the meta-RNA program failed to detect only 6 out of 63,325 high quality 16S rDNA sequences downloaded from the RDP database. We performed an evaluation of such sequences (that were observed to be missed by the meta-RNA program) in the following manner. Using ClustalW [22], we generated a multiple sequence alignment and the corresponding trees indicating the relative distances between 16S rDNA sequences (missed by meta-RNA) and representative sequences of the corresponding genera. It was observed that 16S rDNA sequences missed by meta-RNA are relatively distant from other representative sequences belonging to the same genera. For instance, the 16S rDNA sequence (Sequence Id: S001045291) belonging to *Mycobacterium sp.* 1764, was observed to be highly distinct as compared to 16S rDNA sequences of other species belonging to genus Mycobacterium. The resulting tree (generated using ClustalW) for these sequences is shown in Figure 2. In this figure, sequence S001045291 (belonging to *Mycobacterium sp.* 1764) is observed to be placed at a relatively higher distance from the other 16S rDNA sequences belonging to genus Mycobacterium. Given that the meta-RNA program uses universal HMM models (one each for Bacteria and Archaea) for predicting 16S rDNA fragments, it is expected that the meta-RNA program will miss such 16S rDNA sequences having deviant sequence architectures.

## Conclusions

The paper presents an algorithmic approach that can rapidly identify probable 16S rDNA sequences from metagenomic sequence data sets typically constituted of millions of sequences. The detection sensitivity of this algorithmic approach has been validated using simulated sequence data sets generated using four different sequencing technologies (with sequence lengths ranging from 100 bp to 800 bp). Validation results, even using simulated metagenomic data sets (wherein 16S rDNA sequences originate from entirely new taxonomic clades), indicate high detection sensitivity by the approach discussed in this paper. Furthermore, the i-rDNA algorithm is able to process a million metagenomic sequences in less than an hour.

In addition to the speed of execution and the high detection sensitivity, the utility of the approach discussed in this paper would be immense since it would bypass the entire experimental step of primer-based rDNA amplification, cloning and sequencing. This would result in drastically reducing the cost, time and human efforts invested in all metagenomic projects.

## Methods

### (A) Clustering microbial DNA sequences

DNA sequences from microbial genomes can be clustered based on compositional characteristics such as oligonucleotide usage patterns. For this purpose, fna files (which contain entire genome sequences) corresponding to 237 completely sequenced microbial genomes (one representative from each genera) were downloaded from NCBI (http://ncbi.nlm.nih.gov/). Each genome was split into 1,000 base pair fragments. Frequencies of all possible tetra-nucleotides were computed for each fragment and stored as 256 dimensional vectors. Using k-means clustering approach [23], vectors corresponding to each of these fragments generated from all the microbial genomes were clustered. The Manhattan distance (L1 norm) between individual vectors was used as the similarity measure for clustering. Once the clustering process was completed, vectors corresponding to the centroid of each individual cluster (i.e cluster centroids) were computed and stored.

### (B) Identification of ‘probable rDNA clusters’

For this purpose, 63,325 high quality 16S rDNA sequences were downloaded from the RDP database [24]. For every sequence, a vector representing the frequencies of all 256 tetra-nucleotides was generated. The Manhattan distance of each vector to all pre-computed 'cluster centroids' was obtained. Clusters having the least distance with each individual vector were identified. This process was repeated using vectors corresponding to each of the 63,325 16S rDNA sequences. The frequency with which each cluster was picked up by these sequences was calculated. Clusters having the highest frequency (picked up by a minimum of 10,000 16S rDNA sequences) were identified and tagged as 'probable 16S rDNA clusters'.

**Figure 3:**
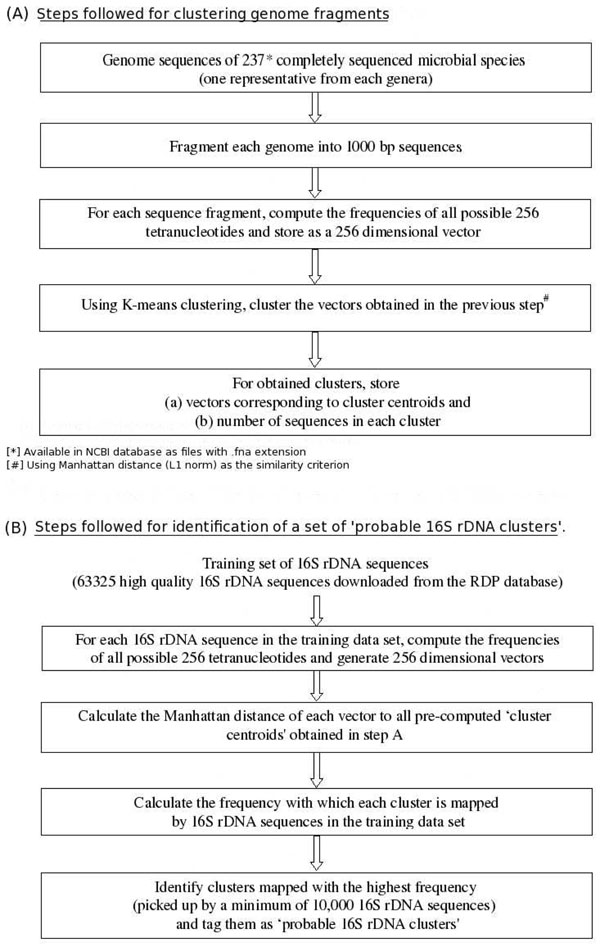
**Pre-processing steps of the i-rDNA approach.** A flowchart illustrating various steps for (A) clustering genomic fragments and (B) identification of ‘probable rDNA clusters’

A flowchart illustrating various steps for (A) clustering genomic fragments and (B) identification of ‘probable rDNA clusters’ is provided in Figure 3.

### (C) Determining optimal threshold values for ‘cumulative sequence count’ and ‘overlap percentage’

There are two run-time values needed by the i-rDNA algorithm. First is the optimal number of closest clusters to be identified for a given query sequence. Second is a value for 'overlap percentage' between the closest clusters and the pre-tagged 'probable 16S rDNA clusters'. The objective was to find the right combination of these values to ensure that the i-rDNA algorithm achieves highest detection sensitivity in the least possible time. For this purpose, 55 genomes (listed in Additional File 2) were used for generating four training data sets each having 550,000 sequences. These 55 organisms were chosen carefully to ensure that there is minimal taxonomic overlap between the organisms in the training and the validation data sets. The taxonomic similarity status between the 112 organisms of the validation sets and the 55 organisms in the training sets is given in Additional File 1.

The i-rDNA algorithm was applied on these training data sets using varying values for the above mentioned two parameters. The value of the first parameter (i.e the number of closest clusters to be identified for a given query sequence) was varied by progressively increasing the value of an encoded parameter, which we termed as 'cumulative sequence count'. For a given query sequence, the value of cumulative sequence count is obtained by progressively adding the number of sequences in the identified closest clusters (which are, in turn, pre-sorted in ascending order of their distance to a given query sequence). Therefore, the number of closest clusters identified for a given query sequence increases with increasing value of 'cumulative sequence count'. The i-rDNA algorithm was applied on the four training data sets using varying values of (a) 'cumulative sequence count' (ranging from 20K to 80K sequences) and (b) overlap percentage (ranging between 20% to 80%). For each combination of parameter values, two results (A and B) were noted down. While value 'A' represented the percentage of training data set sequences reported by i-rDNA as 'probable' 16S rDNA fragments, value 'B' represented the percentage of 'true' 16S rDNA fragments found within the subset of sequences reported by i-rDNA as 'probable' 16S rDNA fragment. The idea was to find the right combination of A and B to ensure that the i-rDNA algorithm achieves highest detection sensitivity in the least possible time. In an ideal scenario, the value of A should be the lowest possible, and the value of B should be the highest possible. A low 'A' value will result in only a small subset of query sequences being redirected to meta-rna program for further analysis. At the same time, the right subset of query sequences should also be picked by the i-rDNA program to ensure minimal loss in detection sensitivity (i.e. resulting in a high value for B).

Results of the above experiments are summarized in Additional File 3. The optimal combination for values 'A' and 'B' (for each sequence lengths) are indicated in bold in the respective tables. Values in these tables were also used for computing the true positive and false positive rates for various thresholds of 'cumulative sequence count' and 'overlap percentage'. Using these true positive and false positive rates, receiver operator characteristics (ROC) curves were generated for different data sets. The methodology used for computing the true positive, false positive rates and the corresponding analysis on the generated ROC curves are provided in Additional File 4.

## List of abbreviations used

i-rDNA: identification of ribosomal DNA; MetaSim: Metagenomic sequence Simulator.

## Competing interests

The authors declare that they have no competing interests.

## Authors' contributions

MMH, TSG and SSM have conceived the idea and designed the detailed methodology. SC implemented the algorithm. MMH and SC created validation data sets, carried out detailed validation and testing of the algorithm. MMH, TSG and SSM have analyzed the data and finally drafted the complete paper.

## Supplementary Material

Additional File 1 List of the 112 organisms constituting the simLC, simMC and the simHC data sets along with their representation status with respect to the 55 genomes of training data set. Click here for file

Additional File 2 Llist of of 55 organisms used for generating four different training data sets corresponding to the sequence lengths of Sanger, 454-Titanium, 454-Standard, 454-GS20 sequencing technologies. Click here for file

Additional File 3 The training results obtained using various combinations of 'cumulative sequence count' (20K, 30K 40K, 50K, 60K, 70K, 80K) and 'overlap percentage' (20%, 30%, 40%, 50%, 60%, 70%, 80%). The four tables 3A-D (in this file) show results obtained with Sanger, 454-400, 454-250 and 454-100 training data sets respectively. Click here for file

Additional File 4 Summary of the methodology used for computing the true positive/false positive rates and the corresponding analysis on the generated ROC curves Click here for file
