# Supplementary material for: i-rDNA: alignment-free algorithm for rapid in silico detection of ribosomal gene fragments from metagenomic sequence data sets
Source: BMC Genomics. 2011 Nov 30;12(Suppl 3):S12. doi: 10.1186/1471-2164-12-S3-S12 (PMC3333171; doi:10.1186/1471-2164-12-S3-S12)
Supplement: Additional File 1 — List of the 112 organisms constituting the simLC, simMC and the simHC data sets along with their representation status with respect to the 55 genomes of training data set. [file 1471-2164-12-S3-S12-S1.pdf]

Additional File 1: List of the 112 organisms constituting the simLC, simMC and the simHC data sets along with their representation status* with respect to the 55 genomes of training data set.

Organism Name	Taxonomic status with respect to 55 organisms used for generating training parameters*
Actinobacillus succinogenes 130Z	Class Shared
Alkalilimnicola ehrlichei MLHE-1	Family Shared
Alkaliphillus metalliredigenes UNDEF	Class Shared
Anabaena variabilis ATCC 29413	Superkingdom Shared
Anaeromyxobacter dehalogenans 2CP-C	Phylum Shared
Arthrobacter sp. FB24	Class Shared
Azotobacter vinelandii AvOP	Class Shared
Bacillus cereus NVH391-98	Phylum Shared
Bifidobacterium longum DJO10A	Species Shared
Bradyrhizobium sp. BTAi1	Class Shared
Brevibacterium linens BL2	Class Shared
Burkholderia ambifaria AMMD	Phylum Shared
Burkholderia cenocepacia AU 1054	Phylum Shared
Burkholderia cenocepacia HI2424	Phylum Shared
Burkholderia sp. sp.strain 383	Phylum Shared
Burkholderia vietnamiensis G4	Phylum Shared
Burkholderia xenovorans LB400	Phylum Shared
Caldicellulosiruptor saccharolyticus UNDEF	Order Shared
Chlorobium limicola DSMZ 245(T)	Superkingdom Shared
Chlorobium phaeobacteroides BS1	Superkingdom Shared
Chlorobium phaeobacteroides DSM 266	Superkingdom Shared
Chlorobium vibrioforme f. thiosulfatophilum DSMZ 265(T)	Superkingdom Shared
Chloroflexus aurantiacus J-10-fl	Family Shared
Chromohalobacter salexigens DSM3043	Order Shared
Clostridium beijerincki NCIMB 8052	Class Shared
Clostridium thermocellum ATCC 27405	Class Shared
Crocosphaera watsonii WH 8501	Superkingdom Shared
Cytophaga hutchinsonii ATCC 33406	Phylum Shared
Dechloromonas aromatica RCB	Phylum Shared
Deinococcus geothermalis DSM11300	All Shared
Desulfitobacterium hafniense DCB-2	Class Shared
Desulfovibrio desulfuricans G20	Phylum Shared
Ehrlichia canis Jake	Class Shared

Ehrlichia chaffeensis sapulpa	Class Shared
Enterococcus faecium DO	Phylum Shared
Exiguobacterium UNDEF 255-15	Phylum Shared
Ferroplasma acidarmanus fer1	Order Shared
Frankia sp. CcI3	Class Shared
Frankia sp. EAN1pec	Class Shared
Geobacter metallireducens GS-15	Phylum Shared
Haemophilus somnus 129PT	Class Shared
Jannaschia sp. CCS1	Family Shared
Kineococcus radiotolerans SRS30216	Class Shared
Lactobacillus brevis ATCC 367	Phylum Shared
Lactobacillus casei ATCC 334	Phylum Shared
Lactobacillus delbrueckii bulgaricus ATCC BAA-365	Phylum Shared
Lactobacillus gasseri ATCC 33323	Phylum Shared
Lactococcus lactis cremoris SK11	Phylum Shared
Leuconostoc mesenteroides mesenteroides ATCC 8293	Phylum Shared
Magnetococcus sp. MC-1	Phylum Shared
Marinobacter aquaeolei VT8	Class Shared
Mesorhizobium sp. BNC1	Class Shared
Methanococcoides burtonii DSM6242	Family Shared
Methanosarcina barkeri Fusaro	Genus Shared
Methanospirillum hungatei JF-1	Order Shared
Methylobacillus flagellatus strain KT	Phylum Shared
Moorella thermoacetica ATCC 39073	Family Shared
Nitrobacter hamburgensis UNDEF	Class Shared
Nitrobacter winogradskyi Nb-255	Class Shared
Nitrosococcus oceani UNDEF	Order Shared
Nitrosomonas eutropha C71	Phylum Shared
Nitrosospira multiformis ATCC 25196	Phylum Shared
Nocardioides sp. JS614	Class Shared
Novosphingobium aromaticivorans DSM 12444 (F199)	Family Shared
Oenococcus oeni PSU-1	Phylum Shared
Paracoccus denitrificans PD1222	Family Shared
Pediococcus pentosaceus ATCC 25745	Phylum Shared
Pelobacter carbinolicus DSM 2380	Phylum Shared
Pelobacter propionicus DSM 2379	Phylum Shared
	Superkingdom Shared

Pelodictyon luteolum UNDEF	Superkingdom Shared
Pelodictyon phaeoclathratiforme BU-1 (DSMZ 5477(T))	Phylum Shared
Polaromonas sp. JS666	Superkingdom Shared
Prochlorococcus marinus str. MIT 9312	Superkingdom Shared
Prochlorococcus sp. NATL2A	Superkingdom Share d
Prosthecochloris aestuarii SK413/DSMZ 271(t)	Class Shared
Pseudoalteromonas atlantica T6c	Class Shared
Pseudomonas fluorescens PfO-1	Class Shared
Pseudomonas putida F1	Class Shared
Pseudomonas syringae B728a	Class Shared
Psychrobacter arcticum 273-4	Class Shared
Psychrobacter cryopegella UNDEF	Species Shared
Rhodobacter sphaeroides 2.4.1	Phylum Shared
Rhodoferax ferrireducens UNDEF	Class Shared
Rhodopseudomonas palustris BisA53	Class Shared
Rhodopseudomonas palustris BisB18	Class Shared
Rhodopseudomonas palustris BisB5	Class Shared
Rhodopseudomonas palustris HaA2	All Shared
Rhodospirillum rubrum ATCC 11170	All Shared
Rubrobacter xylanophilus DSM 9941	Family Shared
Ruegeria sp. TM1040	Class Shared
Saccharophagus degradans 2-40	Class Shared
Shewanella amazonensis SB2B	Class Shared
Shewanella baltica OS155	Class Shared
Shewanella frigidimarina NCMB400	Class Shared
Shewanella putefaciens UNDEF	Class Shared
Shewanella sp. ANA-3	Class Shared
Shewanella sp. MR-7	Class Shared
Shewanella sp. PV-4	Class Shared
Shewanella sp. W3-18-1	Family Shared
Sphingopyxis alaskensis RB2256	Phylum Shared
Streptococcus suis 89/1591	Phylum Shared
Streptococcus thermophilus LMD-9	Phylum Shared
Sulfurimonas denitrificans DSM 1251	Superkingdom Shared
Synechococcus sp. PCC 7942 (elongatus)	Phylum Shared
Syntrophobacter fumaroxidans MPOB	Class Shared
Syntrophomonas wolfei Goettingen	All Shared

Thermoanaerobacter ethanolicus 39E	Class Shared
Thermobifida fusca YX	Phylum Shared
Thiobacillus denitrificans ATCC 25259	Class Shared
Thiomicrospira crunogena XCL-2	Superkingdom Shared
Trichodesmium erythraeum IMS101	Class Shared
Xylella fastidiosa Dixon	

*All Shared: The training data set includes the sequences from the genome of that particular microbe.
*Species Shared: The training data set is devoid of genomes belonging to the strain of that particular microbe
*Genus Shared: The training data set is devoid of genomes that can share either strain or species with that particular microbe.
*Family Shared: The training data set does not consists of any genomes that belong to either strain or species or genus of that particular microbe.
*Order Shared: The training data set does not consists of any genomes that belong to either strain or species or genus or family of that particular microbe.
*Class Shared: The training data set is devoid of genomes that can share either strain or species or genus or family or order with that particular microbe.
*Phylum Shared: The training data set does not consists of any genomes that belong to either strain or species or genus or family or order or class of that particular microbe.
*Super-kingdom Shared: The training data set does not consists of any genomes that belong to either strain or species or genus or family or order or class of that particular microbe.
