# Supplementary material for: i-rDNA: alignment-free algorithm for rapid in silico detection of ribosomal gene fragments from metagenomic sequence data sets
Source: BMC Genomics. 2011 Nov 30;12(Suppl 3):S12. doi: 10.1186/1471-2164-12-S3-S12 (PMC3333171; doi:10.1186/1471-2164-12-S3-S12)
Supplement: Additional File 2 — Llist of of 55 organisms used for generating four different training data sets corresponding to the sequence lengths of Sanger, 454-Titanium, 454-Standard, 454-GS20 sequencing technologies. [file 1471-2164-12-S3-S12-S2.pdf]

Additional File 2: List of 55 organisms used for generating four different training data sets corresponding to the sequence lengths of Sanger, 454-Titanium, 454-Standard, 454-GS20 sequencing technologies respectively.

S. No.	Organism
1	*Acidimicrobium ferrooxidans DSM 10331*
2	*Acidithiobacillus ferrooxidans ATCC 23270*
3	*Acidobacterium capsulatum ATCC 51196*
4	*Aeromonas hydrophila subsp. hydrophila ATCC 7966*
5	*Akkermansia muciniphila ATCC BAA-835*
6	*Archaeoglobus fulgidus*
7	*Bacteroides vulgatus ATCC 8482*
8	*Bifidobacterium longum*
9	*Caulobacter sp. K31*
10	*Cenarchaeum symbiosum A*
11	*Coraliomargarita akajimensis*
12	*Deferribacter desulfuricans*
13	*Deinococcus geothermalis DSM 11300*
14	*Dichelobacter nodosus VCS1703A*
15	*Dictyoglomus turgidum DSM 6724*
16	*Fibrobacter succinogenes subsp. succinogenes S85*
17	*Flavobacterium psychrophilum JIP02/86*
18	*Gemmatimonas aurantiaca T-27*
19	*Hahella chejuensis KCTC 2396*
20	*Haloarcula marismortui ATCC 43049*
21	*Halorhodospira halophila SL1*
22	*Halothermothrix orenii H 168*
23	*Herpetosiphon aurantiacus ATCC 23779*
24	*Ignicoccus hospitalis KIN4/I*
25	*Legionella pneumophila str. Corby*
26	*Leptotrichia buccalis DSM 1135*
27	*Methanobrevibacter smithii ATCC 35061*
28	*Methanococcus aeolicus Nankai-3*
29	*Methanoculleus marisnigri JR1*
30	*Methanopyrus kandleri*
31	*Methanosarcina acetivorans*
32	*Methylococcus capsulatus str. Bath*
33	*Natranaerobius thermophilus JW/NM-WN-LF*
34	*Nitrosopumilus maritimus SCM1*
35	*Parvularcula bermudensis HTCC2503*
36	*Pirellula staleyi DSM 6068*
37	*Pyrobaculum arsenaticum DSM 13514*
38	*Rhodobacter sphaeroides ATCC 17025*

39	*Rhodospirillum rubrum ATCC 11170*
40	*Roseiflexus castenholzii DSM 13941*
41	*Rubrobacter xylanophilus DSM 9941*
42	*Salinibacter ruber DSM 13855*
43	*Slackia heliotrinireducens DSM 20476*
44	*Solibacter usitatus Ellin6076*
45	*Sphaerobacter thermophilus DSM 20745*
46	*Sphingomonas wittichii RW1*
47	*Sulfolobus acidocaldarius DSM 639*
48	*Thermoanaerobacter pseudethanolicus ATCC 33223*
49	*Thermococcus onnurineus NA1*
50	*Thermodesulfovibrio yellowstonii DSM 11347*
51	*Thermomicrobium roseum DSM 5159*
52	*Thermoplasma acidophilum*
53	*Thermus thermophilus HB27*
54	*uncultured methanogenic archaeon RC-I*
55	*Vibrio cholerae*
