# Supplementary material for: i-rDNA: alignment-free algorithm for rapid in silico detection of ribosomal gene fragments from metagenomic sequence data sets
Source: BMC Genomics. 2011 Nov 30;12(Suppl 3):S12. doi: 10.1186/1471-2164-12-S3-S12 (PMC3333171; doi:10.1186/1471-2164-12-S3-S12)
Supplement: Additional File 3 — The training results obtained using various combinations of 'cumulative sequence count' (20K, 30K 40K, 50K, 60K, 70K, 80K) and 'overlap percentage' (20%, 30%, 40%, 50%, 60%, 70%, 80%). The four tables 3A-D (in this file) show results obtained with Sanger, 454-400, 454-250 and 454-100 training data sets respectively. [file 1471-2164-12-S3-S12-S3.pdf]

Additional File 3: Training results obtained using various combinations of 'cumulative sequence count' (20K, 30K 40K, 50K, 60K, 70K, 80K) and 'overlap percentage' (20%, 30%, 40%, 50%, 60%, 70%, 80%). The four tables A-D show results obtained with Sanger, 454-400, 454-250 and 454-100 training data sets respectively.

Note:
Cumulative sequence count: The total number of sequences in a set of clusters identified as having the least distance

Overlap percentage: The percentage of overlap between the set of clusters (identified as compositionally closest to a

(A) Sanger training data set

Total Number of sequences: 550,000 (10,000 reads each from 55 training organisms)
Sequences harboring portions of the 16 rDNA gene: 1411 (0.26%)

Overlap percentage*	A and B values**	Value of cumulative sequence count*** used for identifying a set of clusters closest to the query sequence						
		**20K**	**30K**	**40K**	**50K**	**60k**	**70K**	**80k**
20%	A	31.5	33.8	39.7	39.9	43.2	44.1	46.2
	B	89.9	90.1	90.2	95.1	97.8	98.9	99.9
30%	A	24.5	25.7	28.4	29.1	32.6	33.3	37.4
	B	87.5	87.9	89.1	92.6	94.6	95.1	99.7
40%	A	8.4	9.3	10	**10.2**	13.8	14.3	17.2
	B	83.2	85.6	86.8	**91.4**	91.9	92	93.9
50%	A	7.2	8.8	9.5	9.9	12.3	14	16.8
	B	74.6	78.9	85.9	85.7	91.2	90	88.3
60%	A	6.7	6.9	7.7	8.9	10.1	12.4	15.2
	B	69.9	70	70.4	71.9	72.9	73.5	74.4
70%	A	6.3	6.5	7.2	7.6	9	10.6	14.3
	B	51.4	52.2	55.8	57.3	57.9	58.1	59.6
80%	A	5.5	5.8	7	7.2	8.9	11.8	12
	B	48.7	49.9	50.1	51.2	53.7	54.4	55.1

* Overlap percentage: The percentage of overlap between the set of clusters (identified as compositionally closest to a given query sequence) and the set of pre-tagged 'probable 16S rDNA clusters'

** A represents the percentage of training data set sequences reported by i-rDNA as 'probable' 16S rDNA fragments

** B represents the percentage of 'true' 16S rDNA fragments within the subset of sequences reported by i-rDNA as probable 16S rDNA fragments.

***Cumulative sequence count: The total number of sequences in a set of clusters identified as having the least distance to the vector corresponding to a query sequence.

(B) 454-400 training data set

Total Number of sequences: 550,000 (10,000 reads each from 55 training organisms)
Sequences harboring portions of the 16 rDNA gene: 765 (0.14%)

Overlap percentage*	A and B values**	Value of 'cumulative sequence count' used for identifying a set of clusters closest to the query sequence						
		**20K**	**30K**	**40K**	**50K**	**60k**	**70K**	**80k**
20%	A	33.2	35.5	41.4	41.6	44.9	45.8	47.9
	B	92.4	93.6	94.2	97.3	98	98.8	99.7
30%	A	24.1	26.8	31.9	32.0	34.6	35.3	37.0
	B	90.5	92.9	93.6	96.7	97.8	98.2	99
40%	A	17.9	20.3	22.4	23.6	29.7	30.1	35.2
	B	89.8	92.1	92.7	96	96.7	97.5	98.3
50%	A	12.5	13.7	16.4	**16.5**	20.6	21.3	25.4
	B	86.4	88.1	89.2	**93.1**	93.8	94.6	99.2
60%	A	8.8	9.6	10.5	11.6	14.4	14.9	17.8
	B	61.3	61.5	62.4	65.2	66.2	67.1	69.8
70%	A	6.1	6.4	7.7	7.9	9.7	13	13.4
	B	54.1	55.1	55.6	56.8	59.7	60.1	61.2
80%	A	4.8	5.2	6.3	6.5	7.7	10.5	10.8
	B	45.4	46.3	46.7	47.7	50.2	50.5	51.4

* Overlap percentage: The percentage of overlap between the set of clusters (identified as compositionally closest to a given query sequence) and the set of pre-tagged 'probable 16S rDNA clusters'

** A represents the percentage of training data set sequences reported by i-rDNA as 'probable' 16S rDNA fragments

** B represents the percentage of 'true' 16S rDNA fragments within the subset of sequences reported by i-rDNA as probable 16S rDNA fragments.

***Cumulative sequence count: The total number of sequences in a set of clusters identified as having the least distance to the vector corresponding to a query sequence.

(C) 454-250 training data set

Total Number of sequences: 550,000 (10,000 reads each from 55 training organisms)
Sequences harboring portions of the 16 rDNA gene: 720 (0.13%)

Overlap percentage*	A and B values**	Value of 'cumulative sequence count' used for identifying a set of clusters closest to the query sequence						
		20K	30K	40K	50K	60k	70K	80k
20%	A	34.2	35.2	39.2	41.3	46.4	48.1	55.3
	B	87.1	88.6	90.6	92.9	94.7	97.2	99.8
30%	A	19.5	20.7	23.4	24.1	27.6	28.3	32.4
	B	84.4	85	86.6	90.3	91.7	93.2	96.7
40%	A	14.5	17.3	21.6	22.1	25.7	27	29.4
	B	81.2	82.6	85.1	88.9	89.3	90.1	96.1
50%	A	10.3	11.4	13.7	**13.9**	17.6	18.1	21.8
	B	80.5	81.1	82.3	**85.9**	86.7	86.9	92
60%	A	7.5	8.7	11.4	12.1	15.6	17.9	20.6
	B	74.5	74.9	76.1	79.6	81.6	82.1	86.7
70%	A	4.25	5.9	7.1	8.47	10.7	12.2	14
	B	43.7	44.2	45.7	47.3	48	49.6	52.2
80%	A	3.2	3.9	4.7	5.1	5.7	6.3	7.8
	B	33.7	34.1	34.6	36.2	37.7	37.9	38.7

* Overlap percentage: The percentage of overlap between the set of clusters (identified as compositionally closest to a given query sequence) and the set of pre-tagged 'probable 16S rDNA clusters'

** A represents the percentage of training data set sequences reported by i-rDNA as 'probable' 16S rDNA fragments

** B represents the percentage of 'true' 16S rDNA fragments within the subset of sequences reported by i-rDNA as probable 16S rDNA fragments.

***Cumulative sequence count: The total number of sequences in a set of clusters identified as having the least distance to the vector corresponding to a query sequence.

(D) 454-100 training data set

Total Number of sequences: 550,000 (10,000 reads each from 55 training organisms)
Sequences harboring portions of the 16 rDNA gene: 461 (0.08%)

Overlap percentage*	A and B values**	Value of 'cumulative sequence count' used for identifying a set of clusters closest to the query sequence						
		20K	30K	40K	50K	60k	70K	80k
20%	A	38.3	39.7	39.4	42.8	47.6	49.1	54.6
	B	90.1	91.4	93.6	94.2	96	96.9	99.1
30%	A	33.7	34.1	38	40.5	45.4	47.1	52.3
	B	88.9	89.6	90.9	93	95.4	96.1	98.1
40%	A	18.4	23.3	24.7	25.9	26.7	30.1	33.8
	B	86.3	89.1	89.9	92.4	93.8	95	96.1
50%	A	16.7	17.9	**19.7**	22.8	24.1	26.9	30.6
	B	81.1	83.3	**88.7**	89.1	91.1	92.7	93
60%	A	13.1	15.4	16.9	18.5	20.7	22.3	25.6
	B	71.5	73	73.7	78.4	81.6	82.7	83.2
70%	A	6.8	9.1	10.5	11.9	12.4	13.1	15
	B	38.4	38.2	40.6	42.8	43.7	44.9	45.3
80%	A	4.1	4.9	6.8	7.5	9.9	11	13.2
	B	29.6	30.9	31.4	32.7	33.9	34	35.3

* Overlap percentage: The percentage of overlap between the set of clusters (identified as compositionally closest to a given query sequence) and the set of pre-tagged 'probable 16S rDNA clusters'

** A represents the percentage of training data set sequences reported by i-rDNA as 'probable' 16S rDNA fragments

** B represents the percentage of 'true' 16S rDNA fragments within the subset of sequences reported by i-rDNA as probable 16S rDNA fragments.

***Cumulative sequence count: The total number of sequences in a set of clusters identified as having the least distance to the vector corresponding to a query sequence.
