# Supplementary material for: i-rDNA: alignment-free algorithm for rapid in silico detection of ribosomal gene fragments from metagenomic sequence data sets
Source: BMC Genomics. 2011 Nov 30;12(Suppl 3):S12. doi: 10.1186/1471-2164-12-S3-S12 (PMC3333171; doi:10.1186/1471-2164-12-S3-S12)
Supplement: Additional File 4 — Summary of the methodology used for computing the true positive/false positive rates and the corresponding analysis on the generated ROC curves [file 1471-2164-12-S3-S12-S4.pdf]

Determining optimal threshold values for ‘cumulative sequence count’ and 'overlap percentage

The current document describes the methodology adopted for finding the optimal thresholds for ‘cumulative sequence count' and 'overlap percentage' using ROC curves. For each of the four training data sets, i-rDNA program was executed using various pairs of thresholds for cumulative sequence count and overlap percentage. For each pair of thresholds, the True Positive Rate and the False Positive Rates were determined using values in Additional File 3

For each pair of thresholds (of cumulative sequence count and overlap percentage), the True Positives, the False Positives, the True Negatives and the False Negatives were calculated as follows.

TRUE POSITIVES (TP): Number of true 16S rDNA fragments that were identified as 'probable 16S rDNA fragment' by i-rDNA.

FALSE POSITIVES (FP): Number of non-16S rDNA fragments that were identified as 'probable 16S rDNA fragment' by i-rDNA.

TRUE NEGATIVES (TN): Number of non-16S rDNA fragments that were identified as 'non-16S rDNA fragment' by i-rDNA.

False NEGATIVES (FN): Number of true 16S rDNA fragments that were identified as 'non-16S rDNA fragment' by i-rDNA.

Subsequently, the True Positive Rate (TPR) and the False Positive Rate (FPR) was calculated using the following formulae.

TPR = TP / (TP + FN)

FPR = FP / (FP + TN)

For each of the training data sets, the performance of i-rDNA (in terms of TPR and FPR) was evaluated using a range of threshold pairs (i.e., cumulative sequence count and overlap percentage). The TPR v/s FPR values obtained (for each of the thresholds) were plotted as ROC curves. These ROC curves are provided below. The optimal values for cumulative sequence count and overlap percentage are also indicated (for each data set).

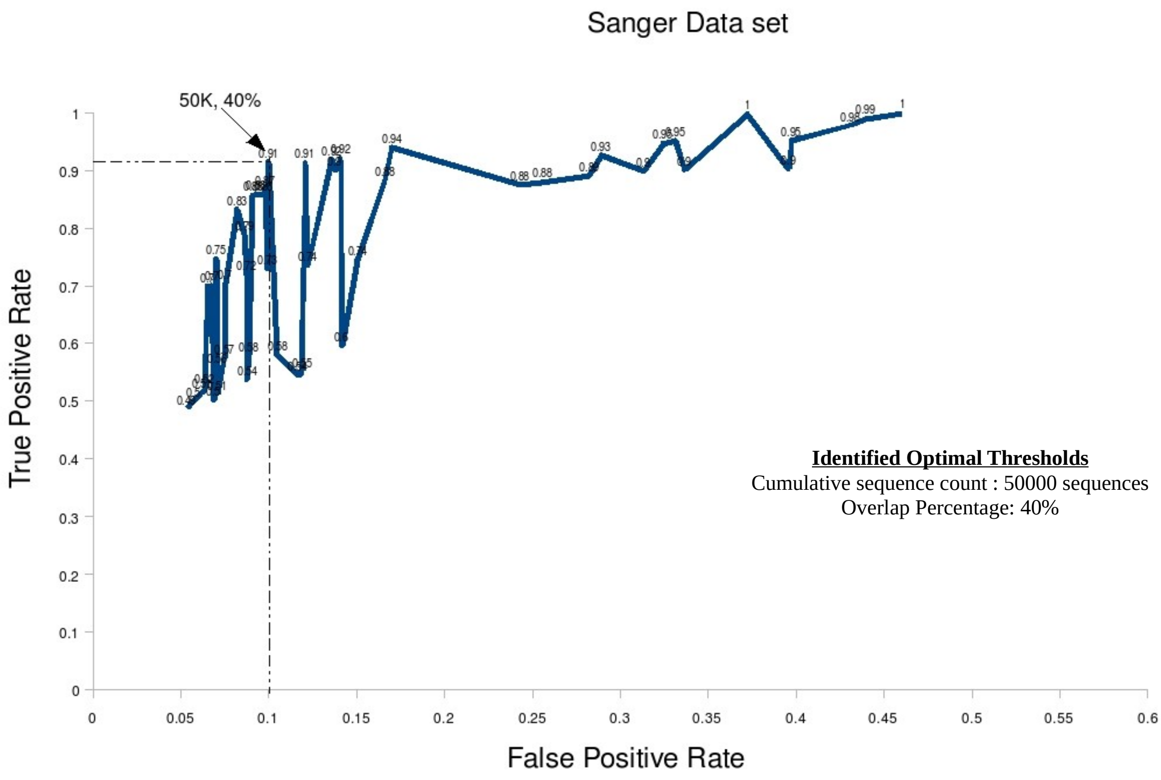
Sanger Data set
50K, 40%
True Positive Rate
False Positive Rate
Identified Optimal Thresholds
Cumulative sequence count : 50000 sequences
Overlap Percentage: 40%

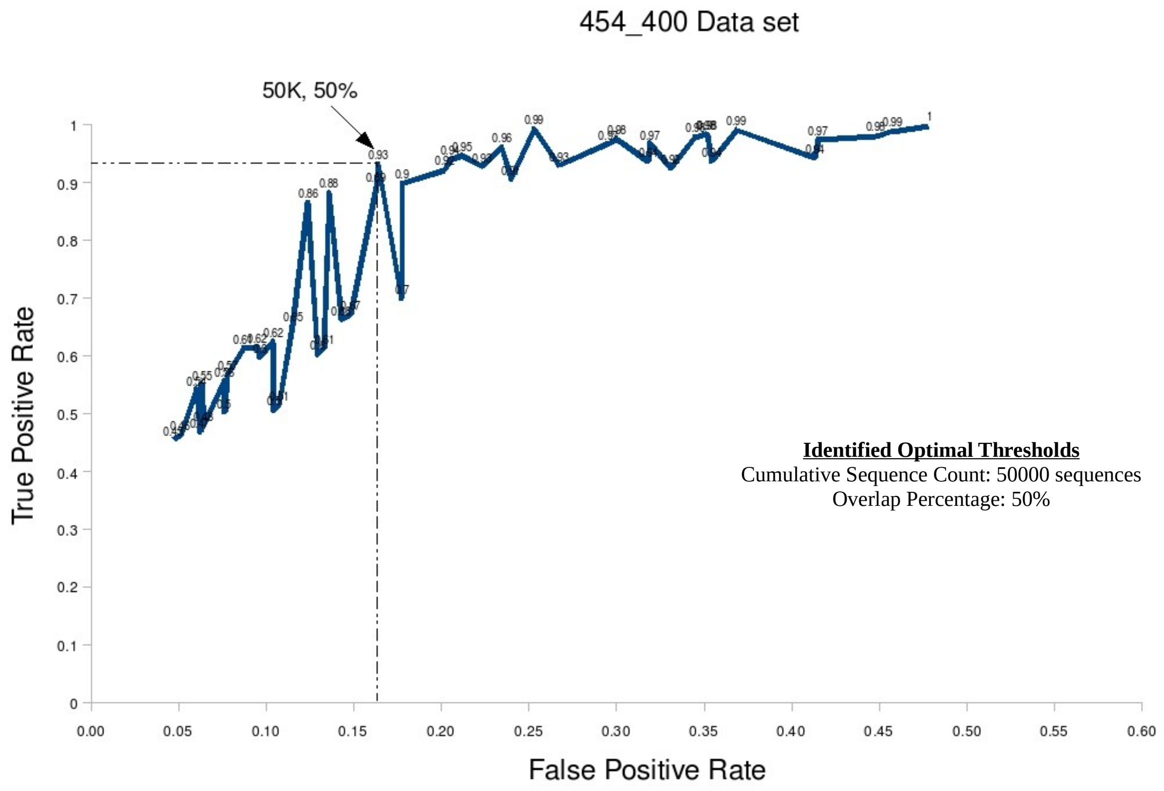
454_400 Data set
50K, 50%
True Positive Rate
False Positive Rate
Identified Optimal Thresholds
Cumulative Sequence Count: 50000 sequences
Overlap Percentage: 50%

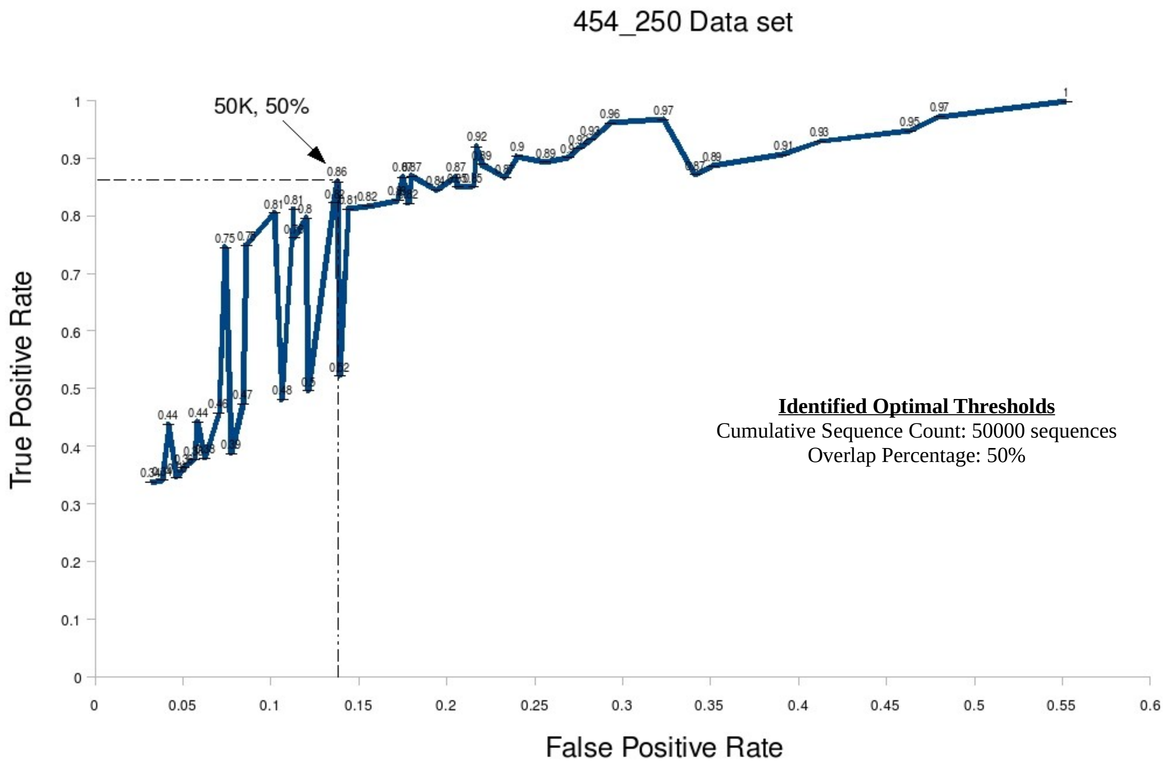
454_250 Data set
50K, 50%
True Positive Rate
False Positive Rate
Identified Optimal Thresholds
Cumulative Sequence Count: 50000 sequences
Overlap Percentage: 50%

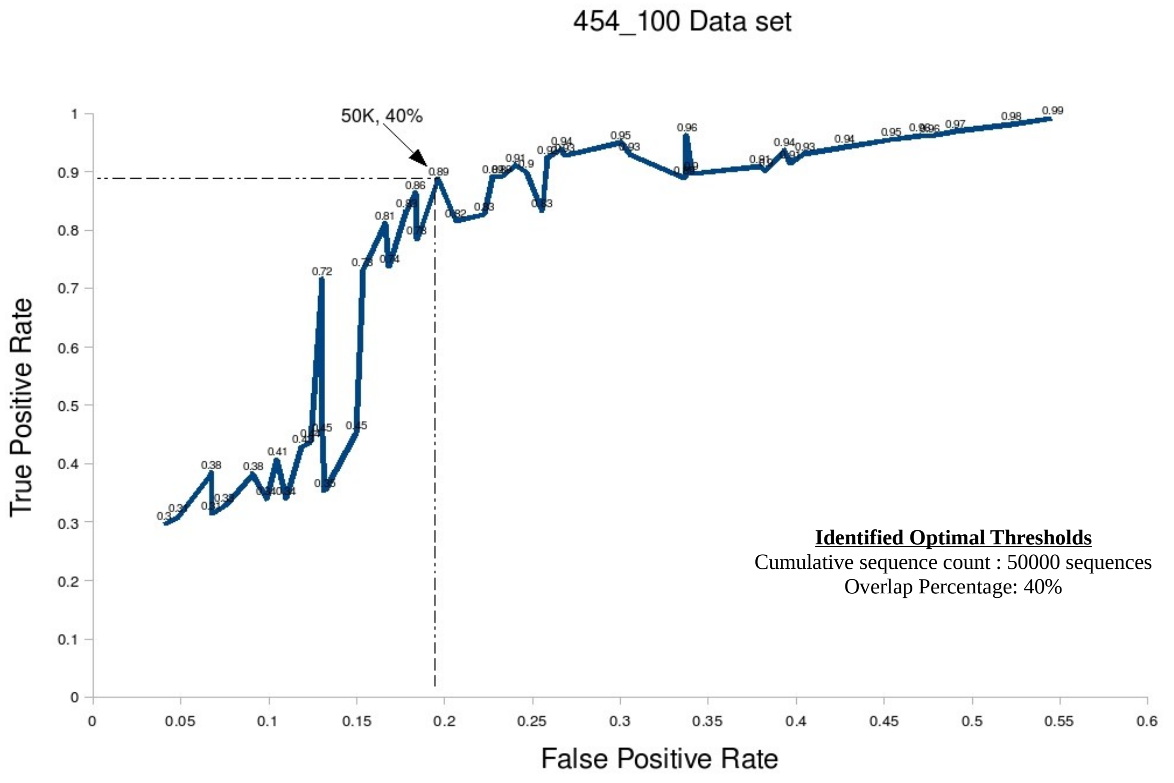
454_100 Data set
50K, 40%
True Positive Rate
False Positive Rate
Identified Optimal Thresholds
Cumulative sequence count : 50000 sequences
Overlap Percentage: 40%

ANALYSIS

For the four training data sets, the points corresponding to the 'optimal thresholds' (for cumulative sequence count and overlap percentage) are indicated for each of the ROC curves illustrated above. The values of optimal thresholds identified are also indicated.

As observed in the four ROC curves, the set of optimal thresholds identified for each training data set ensure a True Positive Rate of greater than 85%, while limiting the False Positive Rate to less than 15%. For thresholds corresponding to the points that lie to the left of the indicated 'optimal threshold' point, the True Positive Rates (i.e sensitivity) of the i-rDNA program are observed to drop drastically. On the other hand, using thresholds corresponding to points that lie to the right of the 'optimal threshold' point leads to a drastic increase in the false positive rate with only a marginal increase in detection sensitivity.
